# Functional characterization of *MANNOSE-BINDING LECTIN 1*, a G-type lectin gene family member, in response to fungal pathogens of strawberry

**DOI:** 10.1093/jxb/erac396

**Published:** 2022-10-11

**Authors:** Lijing Ma, Zeraye Mehari Haile, Silvia Sabbadini, Bruno Mezzetti, Francesca Negrini, Elena Baraldi

**Affiliations:** Department of Agricultural and Food Science, DISTAL, Alma Mater Studiorum - University of Bologna, Bologna, Italy; Department of Agricultural and Food Science, DISTAL, Alma Mater Studiorum - University of Bologna, Bologna, Italy; Plant Protection Research Division of Melkasa Agricultural Research Center, Ethiopian Institute of Agricultural Research (EIAR), Addis Ababa, Ethiopia; Department of Agricultural, Food and Environmental Sciences, Università Politecnica delle Marche, Ancona, Italy; Department of Agricultural, Food and Environmental Sciences, Università Politecnica delle Marche, Ancona, Italy; Department of Agricultural and Food Science, DISTAL, Alma Mater Studiorum - University of Bologna, Bologna, Italy; Department of Agricultural and Food Science, DISTAL, Alma Mater Studiorum - University of Bologna, Bologna, Italy; University of Trento, Italy

**Keywords:** Anthracnose, fungal disease resistance, B-lectin, *Botrytis cinerea*, *Colletotrichum fioriniae*, *Fragaria × ananassa*, G-type lectin, grey mould, phytohormones, strawberry

## Abstract

The mannose-binding lectin gene *MANNOSE-BINDING LECTIN 1* (*MBL1*) is a member of the G-type lectin family and is involved in defense in strawberry (*Fragaria* × *ananassa*). Genome-wide identification of the G-type lectin family was carried out in woodland strawberry, *F. vesca*, and 133 G-lectin genes were found. Their expression profiles were retrieved from available databases and indicated that many are actively expressed during plant development or interaction with pathogens. We selected *MBL1* for further investigation and generated stable transgenic *FaMBL1*-overexpressing plants of *F.* ×*ananassa* to examine the role of this gene in defense. Plants were selected and evaluated for their contents of disease-related phytohormones and their reaction to biotic stresses, and this revealed that jasmonic acid decreased in the overexpressing lines compared with the wild-type (WT). Petioles of the overexpressing lines inoculated with *Colletotrichum fioriniae* had lower disease incidence than the WT, and leaves of these lines challenged by *Botrytis cinerea* showed significantly smaller lesion diameters than the WT and higher expression of *CLASS II CHITINASE 2-1*. Our results indicate that *FaMBL1* plays important roles in strawberry response to fungal diseases caused by *C. fioriniae* and *B. cinerea*.

## Teaser

*FaMBL1*plays a role in strawberry defense against fungal pathogens, and transgenic octoploid strawberries overexpressing *FaMBL1*are less susceptible to anthracnose and grey mould.

## Introduction

Strawberry (*Fragaria × ananassa*) is an economically important fruit worldwide and is considered a model plant system for the *Rosaceae*. It is susceptible to a large number of pathogens including the *Colletotrichum acutatum* species complex (anthracnose) and *Botrytis cinerea* (grey mould), which cause enormous economic losses (Guidarelli *et al.*, 2011; Petrasch *et al.*, 2019). Although both fungi can infect the fruits at both unripe and ripe stages, the symptoms appear only on red ripe fruits whilst on white unripe fruits the pathogens become quiescent. Transcriptome analysis of white and red fruits inoculated with *C. acutatum* have shown that a mannose-binding lectin gene, *FaMBL1* (GenBank accession no. KF962716) is the most up-regulated gene in resistant white fruit (Guidarelli *et al.*, 2011). Transient transformation to silence *FaMBL1* results in white fruit with an increased susceptibility to *C. acutatum* (Guidarelli *et al.*, 2014).

The protein encoded by *FaMBL1* is composed of an N-terminal signal peptide, a *Galanthus nivalis* agglutinin-related lectin (GNA) domain, and a Pan-apple (PAN) domain. The GNA domain is the characteristic domain of G-type lectin, which is an important family of plant lectins that have affinity to mannose or mannose-containing N-glycans (Barre *et al.*, 2001). Due to their ability to recognize and bind mannose, G-type lectins have important functions in plant growth and defense. The roles of G-type lectins in resistance to insects, fungi, and bacteria have been described; for example, the expression of G-type lectin genes in potato (Down *et al.*, 1996), maize (Wang *et al.*, 2005), and wheat (Miao *et al.*, 2011) increases resistance to aphids by inhibiting their development and decreasing fecundity. The pepper G-type lectin genes *CaMBL1* and *CaGLP1* have also been found to be involved in defense against *Xanthomonas campestris* pv *vesicatoria* and are required for plant cell death and defense signaling (Hwang and Hwang, 2011; Kim *et al.*, 2015). The G-type lectin gene *Pi-d2* from rice cv. Digu provides resistance to *Magnaporthe grisea*. The transfer of *Pi-d2* from the resistant Digu to the susceptible cv. TP309 confers the latter with resistance to *Magnaporthe grisea* (Chen *et al.*, 2006). Interestingly, the difference between the native Pi-d2 protein forms in Digu versus TP309 is only a single amino acid change (Ile441Met) in the transmembrane (TM) domain. This suggests that the TM domain plays an important role in the ligand recognition and signal transduction (Chen *et al.*, 2006). Another G-type lectin, LORE, from Arabidopsis is composed of GNA, S-locus glycoprotein, PAN, transmembrane, and kinase domains and plays a role in plant innate immunity via sensing the lipopolysaccharide (LPS) of *Pseudomonas* and *Xanthomonas* (Ranf *et al.*, 2015). Accordingly, Arabidopsis Col-0 plants pre-treated with *Pseudomonas* LPS are more resistant to subsequent infection with *Pseudomonas*. To determine the role of different domains, Ranf *et al.* (2015) produced the mutants *lore-1* and *lore-2* by protein truncation after the S-locus glycoprotein domain and substitution of an amino acid (Gly391Glu) in the PAN domain, respectively, and found that LPS-induced resistance was lost in both.

It is well known that the balance of hormonal crosstalk strongly influences the outcome of plant–pathogen interactions (Robert-Seilaniantz *et al.*, 2011), and the contribution of lectins to plant resistance seems to be consistently displayed in a phytohormone-dependent manner (Bonaventure, 2011; Gilardoni *et al.*, 2011; Hwang and Hwang, 2011).

Despite the clear importance of G-type lectins in plant defense responses, there have been few studies examining their expression strawberry (Martínez Zamora *et al.*, 2008; Ma *et al.*, 2021). Recently, with the release of the updated genome annotation and comprehensive gene expression atlas of woodland strawberry, *F. vesca* (Li *et al.*, 2019), reliable data have become available for genome-wide analysis of G-type lectin genes in strawberry. In this study, we used this genome annotation together with the available transcriptome data to identify the G-lectin gene family members in this species and to analyse their domain arrangements and expression profiles. The results implied the great potential of many G-lectin members in strawberry defense responses and provided the basis for functional characterization of *FaMBL1* in cultivated strawberry. To gain insights into the effects of this gene on plant defense, we generated and characterized genetically transformed plants overexpressing *FaMBL1*.

## Materials and methods

### Genome-wide analysis of G-type lectin genes in *F. vesca*

#### Identification and domain organization of G-type lectins

To identify G-type lectin genes, the GNA domain of *FvH4_3g18380* (homolog of *FaMBL1*) was used as the query for a BLASTp search in the Genome Database for Rosaceae (GDR; https://www.rosaceae.org/) (Jung *et al.*, 2019). The *Fragaria vesca* Whole Genome v4.0.a2 database was also used (https://www.rosaceae.org/species/fragaria_vesca/genome_v4.0.a2). Results with E-values <1 × 10^–6^ were considered as G-type lectin protein candidates. With the same settings, a second BLASTp was conducted using a number of variable GNA domains from G-lectin proteins found in the first BLASTp search: FvH4_1g23370, FvH4_2g12390, FvH4_2g14250, FvH4_2g26490, FvH4_2g29050, FvH4_2g33830, FvH4_3g03230, FvH4_3g03301, FvH4_3g03410, FvH4_3g03430, FvH4_3g06140, FvH4_3g15930, FvH4_3g18370, FvH4_3g21270, FvH4_3g43440, FvH4_4g02170, FvH4_5g31680, FvH4_6g00300, FvH4_6g12870, FvH4_6g44106. The domains of each candidate gene were checked manually using the InterProScan website (https://www.ebi.ac.uk/interpro/search/sequence/) (Quevillon *et al.*, 2005). The transmembrane domain was checked using TMHMM Server v. 2.0 (https://dtu.biolib.com/DeepTMHMM) (Krogh *et al.*, 2001).

#### Chromosome location of G-type lectins

Visualization of the chromosome locations of the G-lectin genes was accomplished using MapGene2Chromosome v.2.0 (http://mg2c.iask.in/mg2c_v2.0/) (Chao *et al.*, 2015). The coordinates of the G-lectin genes on the strawberry genome were obtained from the GDR website (*F. vesca* v4.0.a2).

#### Expression profiles of G-type lectin genes

Expression profiles for the *F. vesca* G-lectin genes were retrieved from the supplementary material provided by Li *et al.* (2019). The expression levels of the genes in different tissues (flowers, fruits at different developmental stages, seedlings, leaves, meristems, and roots) were used to construct a heatmap using the the R package, ComplexHeatmap (Gu *et al.*, 2016).

To infer the expression of G-lectin genes upon pathogen infection, we used the transcriptome profiles of *F*. *×ananassa* infected by *Botrytis cinerea* (Xiong *et al.*, 2018; Haile *et al.*, 2019), and of *F. vesca* infected by *Phytophthora cactorum* (Toljamo *et al.*, 2016) and by *Podosphaera aphanis* (Jambagi and Dunwell, 2015) . In addition, we also used transcriptome profiles of *F.×ananassa* after cold stress (Zhang *et al.*, 2019) and after preharvest application of benzothiadiazole and chitosan (Landi *et al.*, 2017) to obtain G-lectin gene expression profiles in different tissues. For this, the G-lectin genes from the *F.×ananassa* transcriptome datasets were converted to their *F. vesca* orthologs.

### Genetic transformation and characterization of *FaMBL1* in *F*. ×*ananassa*

#### Transgenic plants and droplet digital PCR

Genetically transformed plants overexpressing *FaMBL1* were produced and their copy numbers of the transgene were determined by droplet digital PCR (ddPCR) (Ma *et al.*, 2021). Briefly, the full-length sequence of *FaMBL1* was cloned into the pK7WG2 vector (https://gatewayvectors.vib.be/) and the resulting construct overexpressing *FaMBL1* was checked by PCR, restriction enzyme digestion, and sequencing of the PCR product (Ma *et al.*, 2021). The resulting vector, *35S::FaMBL1*, was introduced into *Agrobacterium tumefaciens* strain EHA105 using the freeze–thaw shock method (Weigel and Glazebrook, 2006) to generate transgenic plants. Expanded leaves from 3-week-old *in vitro* elongated strawberry shoots (*F.* ×*ananassa* cv. Sveva) were used as starting explants for the genetic transformation trial, following the protocol developed by Cappelletti *et al.* (2015). The transformed lines were screened by selective media and specific amplicons via PCR (Ma *et al.*, 2021). Three lines (18F6G1, 19F2G1, and 27F8G1) were selected and used for this study.

ddPCR was performed to determine the copy number of the target gene in each transgenic line using the QX100™ Droplet Digital PCR System (Bio-Rad), following the process described in our previous study (Ma *et al.*, 2021). The copy numbers of overexpressing vectors obtained in this study are relative values. The overexpressing lines with different copy numbers were subsequently propagated through stolons in a greenhouse to obtain 30 plants for each transformed line, which were then used in the subsequent experiments. The plants were grown in pots containing soil, with one plant per pot, under natural daylight.

#### RNA extraction, cDNA synthesis, and quantitative PCR

Young leaves, stolons, and petioles (150 mg per sample) of similar growth stages were sampled from mature transgenic and wild-type (WT) plants and were used for RNA extraction following a rapid CTAB method (Gambino *et al.*, 2008). The obtained RNA was treated using a TURBO DNA-free™ Kit (Invitrogen) for removal of residual DNA. The purified RNA was quantified using a NanoDrop 1000 Spectrophotometer (Thermo Scientific) and RNA integrity was checked on an agarose gel. First-strand cDNA was synthesized using an ImProm-II™ Reverse Transcription System (Promega).

Quantitative PCR (qPCR) was conducted to evaluate the expression level of *FaMBL1* of the overexpressing lines and the WT strawberry plants, according to the Minimum Information for Publication of Quantitative Real-Time PCR Experiments (MIQE) guidelines (Bustin *et al.*, 2009). The housekeeping gene *ELONGATION FACTOR 1a* (*FaEF1a*, accession no. BK009992.1) was used as the reference (Guidarelli *et al.*, 2011). The primers *FaMBL1*_CUR FW and *FaMBL1*_CUR rev were used to amplify *FaMBL1* gene. All primers used in this study are listed in Supplementary Table S1 and were designed using the Primer3 software (http://bioinfo.ut.ee/primer3/. qPCR was performed using three biological replicates and two technical replicates, using SYBR™ Green PCR Master Mix (Thermo Fisher) and a Mx3000P qPCR System (Agilent). Gene expression was calculated using a standard curve and normalized by *FaEF1a*.

#### Hormone profiling of *FaMBL1*-overexpressing plants

In order to investigate the contents of defense-related phytohormones in the *FaMBL1*-overexpressing lines and the WT, phytohormones were extracted as described by Glauser *et al.* (2014) from leaves propagated from stolons. A young and fully expanded leaf was sampled from each of three plants per line and pooled together as one replicate, and a total of three replicates were used for each line. The samples were frozen immediately in liquid nitrogen and ground into fine powder. Jasmonic acid (JA), jasmonoyl-isoleucine (JA-Ile), salicylic acid (SA), abscisic acid (ABA), and indole-3-acetic acid (IAA) were measured using ultra-high-pressure liquid chromatography-tandem mass spectrometry (UHPLC-MS/MS), according to the protocol described by Glauser *et al.* (2014), with 50 mg of tissue powder being used for IAA and 100 mg being used for the others. The hormone concentrations were calculated based on calibration curves. Five calibration points were set at 0.1, 0.5, 2, 20, and 100 ng ml^−1^, and the solutions of calibration points and leaf samples contained each of the labeled internal standards at concentrations of 10 ng ml^−1^ for d5-JA, d6-ABA, d6-SA, ^13^C6-JA-Ile, and 1 μg ml^−1^ for d5-IAA.

#### Evaluation of susceptibility of *FaMBL1*-overexpressing plants to fungal diseases

The susceptibility of *FaMBL1*-overexpressing lines to anthracnose was evaluated using *Colletotrichum fioriniae*, which belongs to the *C. acutatum* species complexes (Damm *et al.*, 2012), and it had previously been found to be the most aggressive species to strawberry stocked in our lab. It was cultured on potato dextrose agar (PDA, in Petri dishes). After 10 d, conidia were harvested in distilled water and filtered through three layers of medical gauze. Using a hemocytometer the concentration was adjusted to 2 × 10^4^ conidia ml^−1^ for use as the inoculum. Plants propagated from stolons were used for *C. fioriniae* inoculation. Five petioles of similar growth stage were used for each overexpressing line and the WT. Leaves were removed, and both ends of the petioles were then embedded in moist tissue paper to reduce desiccation. A tiny wound was made on each petiole using a sterilized needle and a 10-µl droplet of inoculum was placed on top of the wound. All inoculated petioles were put in a large plastic tray with moist tissue paper on the bottom. The tray was kept on a bench in the laboratory at room temperature. The disease incidence was recorded at 6 days post-inoculation (dpi) and calculated as the number of petioles with anthracnose symptoms divided by the total number of petioles treated with *C. fioriniae*, and expressed as a percentage. The disease incidence was determined as the mean value of three repeated trials.

The susceptibility of *FaMBL1*-overexpressing lines to *B. cinerea* (isolate B05.10) was determined using conidia harvested from 1-week-old *B. cinerea* cultured on PDA. To stimulate spore germination, a buffer containing 0.5% KH_2_PO_4_ and 1% PDA was prepared and the conidia suspension was mixed with buffer at a ratio of 1:1 (v:v) to get 1 × 10^4^ conidia ml^−1^. The plants used for *B. cinerea* inoculation were the same as those used for *C. fioriniae* inoculation, and young, healthy, and fully expanded leaves of similar growth stages were sampled. One leaf (three leaflets) from each of five different plants per line was sampled and surface-sterilized using 1% sodium hypochlorite for 1 min, and then rinsed in sterilized distilled water for 2 min. The leaves were then separated into leaflets and put in Petri dishes (150 × 15 mm) with moist tissue paper on the bottom. Two 7.5-µl droplets of inoculum were placed on the adaxial side of each leaflet, one on each side of the midrib. Distilled water was sprayed into each Petri dishes to keep the humidity high and the dishes were kept on a laboratory bench at room temperature. The lesion diameters on the leaflets were recorded daily from 3–5 dpi. The rate of increase of lesion diameter was used to indicate the rate of disease progression, and was calculated as (diameter_5dpi_ – diameter_3dpi_)/2. The inoculation trial was repeated three times.

#### Determination of gene expression levels in leaves inoculated with *B. cinerea*

To investigate the molecular responses of the *FaMBL1*-overexpressing lines upon *B. cinerea* inoculation, we used qPCR to determine the expression levels of pathogenesis-related and hormone-synthesis genes. Six leaves of each transgenic line and the WT were subjected to inoculation with either *B. cinerea* as described above or a mock solution using the same volume of the buffer alone (0.25% KH_2_PO_4_ and 0.5% PDA). Individual leaflets were inoculated, then at 1 dpi they were pooled back together as leaves and frozen immediately in liquid nitrogen, and stored at –80°C until use. The expression of *FaMBL1*, *FaPGIP* (EU117215.1), *THAUMATIN-LIKE PROTEIN 1b* (*FaTLP1b*, XM_004287756.2), *PHENYLALANINE AMMONIA-LYASE 1* (*FaPAL1*, KX450226.1), *CLASS II CHITINASE* (*FaChi2-1*, MK301536.1; *FaChi2-2*, MF804503.1), *ALLENE OXIDE SYNTHASE 1* (*FaAOS1*, XM_004291875.2), and *1-AMINOCYCLOPROPANE-1-CARBOXYLATE OXIDASE* (*FaACO*, AY706156.1) was measured by qPCR as described above. The primers used for are listed in Supplementary Table S1.

#### Statistical analysis

Student’s *t*-test or one-way ANOVA followed by Fisher’s LSD test were conducted using the OriginPro 2018 statistical software (https://www.originlab.com/2018).

## Results

### Genome-wide analysis of G-type lectin genes in *F. vesca*

#### Identification and classification

**Fig. 1.**
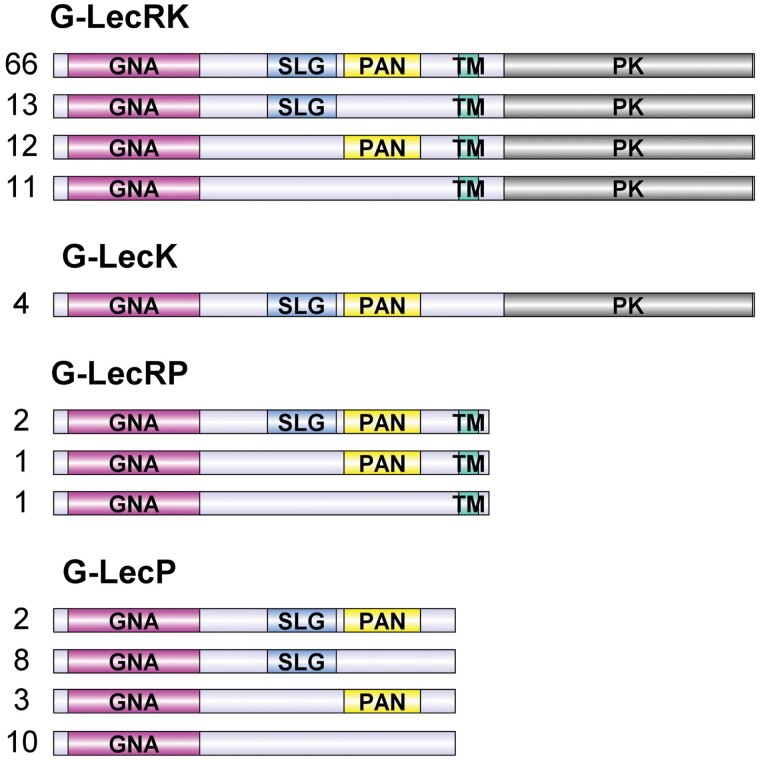
Domain arrangements and classification of G-type lectin proteins in *Fragaria vesca* according to a BLASTp search of the Genome Database for Rosaceae. G-LecRK, G-type lectin receptor kinases; G-LecK, G-type lectin kinases; G-LecRP, G-type lectin receptor proteins; and G-LecP, G-type lectin proteins. GNA, *Galanthus nivalis* agglutinin-related lectin domain; SLG, S-locus glycoprotein domain; PAN, pan-apple domain; PK, protein kinase domain; TM, transmembrane domain. The numbers of corresponding *F. vesca* G-type lectin genes are indicated.

A total of 133 proteins with *Galanthus nivalis* agglutinin (GNA-)related lectin domains were found in *F. vesca*. Among these, 102 containing protein kinase (PK) and transmembrane (TM) domains were classified as G-type lectin receptor kinases (G-LecRKs); 23 lacking both domains were classified as G-type lectin proteins (G-LecPs); four lacking PK but retaining the TM domain were grouped as G-type lectin receptor proteins (G-LecRPs); and four missing the TM domain but containing the PK domain were classified as G-type lectin kinases (G-LecKs). Besides the TM and PK domains, most of the G-type lectins in *F. vesca* also contained domains such as S-locus glycoprotein and/or Pan-apple (PAN) that are involved in self-incompatibility and protein–protein interactions, respectively. Thus, *F. vesca* G-lectins showed multiple domain arrangements (Fig. 1).

For convenience and clarity with regards to the *F. vesca* G-lectin genes, we propose a nomenclature based on the classification of G-lectin genes and their chromosome locations (Supplementary Table S2). According to this scheme, ‘Fve’ at the beginning of the name indicates that the gene is from *F. vesca* (Jung *et al.*, 2015), and then ‘GLRK’, ‘GLRP’, ‘GLP’, and ‘GLK’ represent G-LecRK, G-LecRP, G-LecP, and G-LecK, respectively. The number of the chromosome where the gene is located is then given, followed by a sequential number according to the chromosome location of each type of G-lectin gene on this chromosome. Thus, FvH4_3g18380 (the homolog of *FaMBL1*) becomes *FveGLP3.7*, i.e. it encodes a G-LecP, it is located on chromosome 3, and it is the seventh G-LecP on this chromosome. This nomenclature for *F. vesca* G-type lectins is used hereafter in this paper.

#### Chromosome locations of G-type lectin genes

**Fig. 2.**
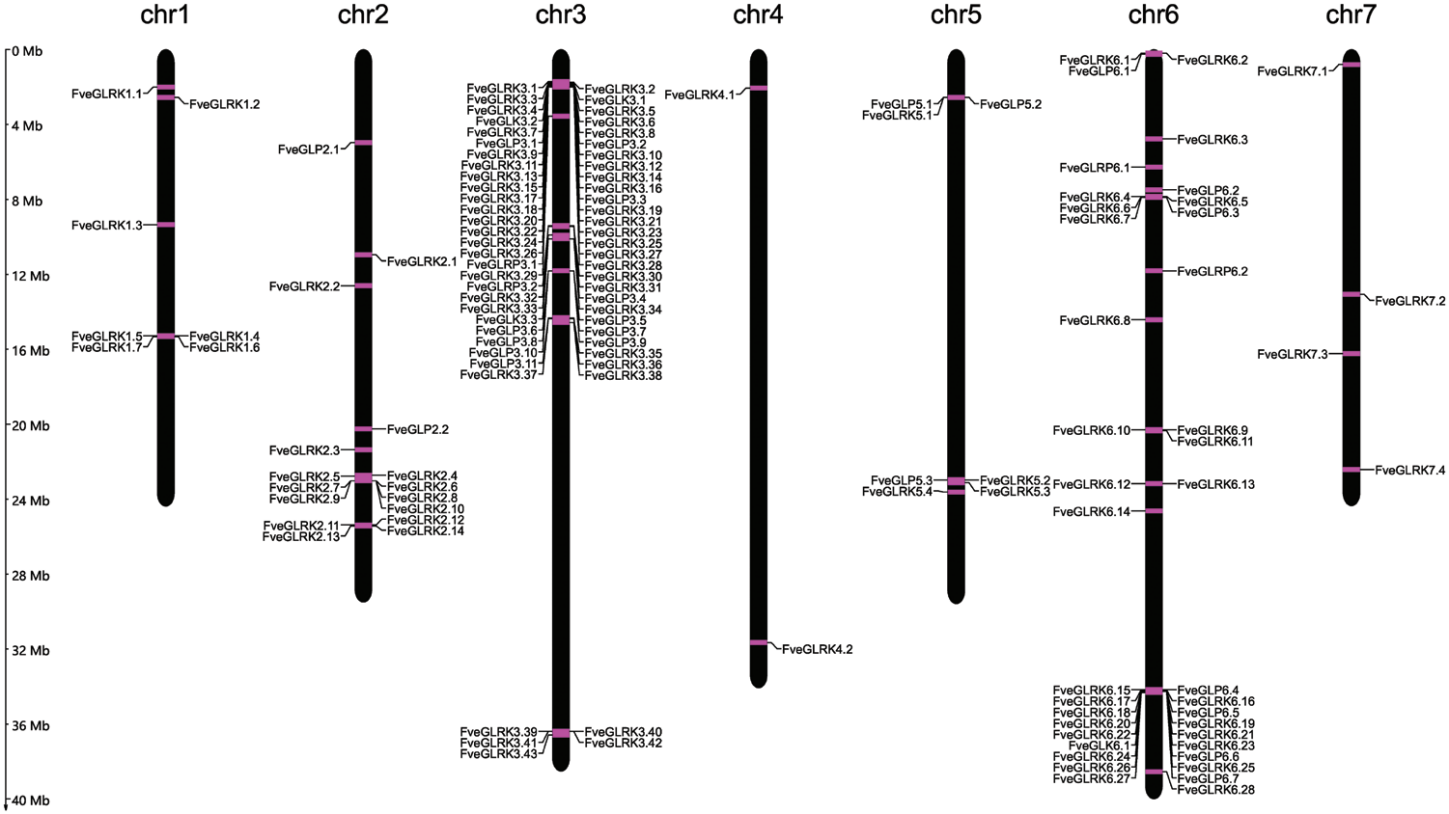
Chromosome localizations of G-type lectin genes in *Fragaria vesca*. The coordinates of the genes on the strawberry genome were obtained from the Genome Database for Rosaceae (F. vesca v4.0.a2).

In order to visualize the chromosome locations, the G-lectin genes were mapped to the *F. vesca* genome (Fig. 2). G-LecRKs were found to be distributed on all the chromosomes, whereas the majority of G-LecRKs were mapped on chromosomes 3 and 6, G-LecPs were mapped on chromosomes 2, 3, 5, and 6, and G-LecRPs were found only on chromosomes 3 and 6.

#### Expression profiles of G-lectin genes based on RNA-seq datasets

**Fig. 3.**
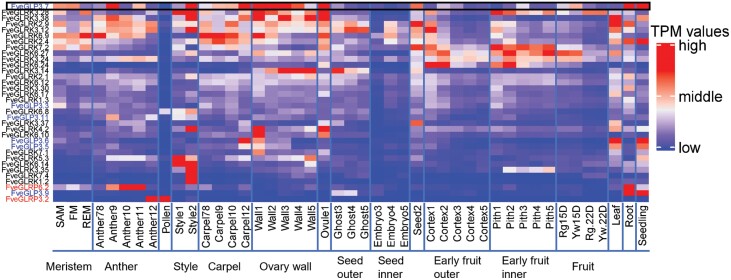
Heatmap of the most expressed G-type lectin genes in *Fragaria vesca*. Expression profiles were retrieved from the database reported by Li *et al.* (2019). G-type lectin receptor kinases (G-LecRKs) are named in black, G-type lectin proteins (G-LecPs) are in blue, and G-type lectin receptor proteins (G-LecRPs) are in red. The expression levels are shown as transcripts per million reads (TPM) and the value for each gene was scaled before constructing the heatmap. The expression profile of *FveGLP3.7* (the homolog of *FaMBL1*) is highlighted by the black border. SAM, shoot apical meristem; FM, flower meristem; REM, receptacle meristem. For other tissues, the numbers following the name indicate the developmental stage, where ‘78’ indicates stages 7–8 for anthers and carpels. Ghost, endosperm and seed coat; Cortex, outer part of the early fruit; Rg.15D, green fruit stage of cv. Ruegen; Yw.15D, green fruit stage of cv. Yellow Wonder 5AF7; Rg.22D, white fruit stage of Ruegen; Yw.22D, white fruit stage of Yellow Wonder 5AF7.The complete heatmap of all the FveG-lectin genes is shown in Supplementary Fig. S1.

The expression profiles of G-lectin genes were analysed in different tissues and at different developmental stages based on an available strawberry RNA-seq dataset (Li *et al.*, 2019). This showed that G-type lectin genes display a wide range of transcription levels, with some genes highly expressed in various tissues including the style, ovary wall, leaves, and roots, while others are completely silenced (Fig. 3; Supplementary Fig. S1). A few genes appeared to be specifically expressed only in one or two tissues, such as *FveGLRP3.2* for example, which was highly expressed in pollen and anthers, but not in the other tissues. In contrast, *FveGLP3.7* (the homolog of *FaMBL1*) showed active expression in many tissues during development, such as in seedlings, roots, and the ovary wall (Fig. 3).

**Fig. 4.**
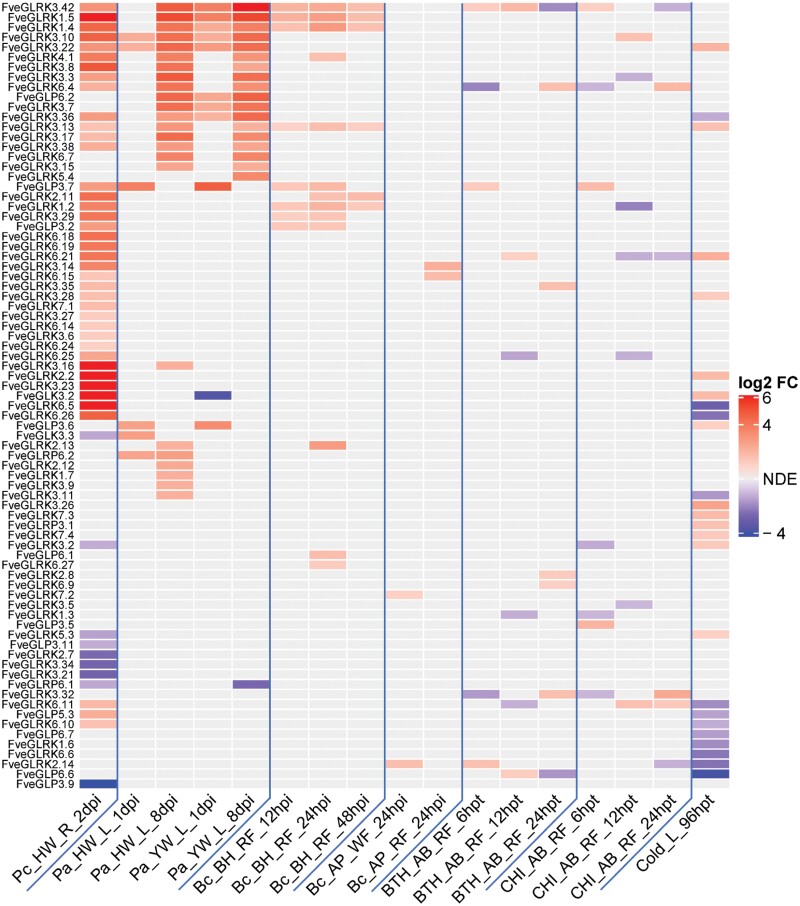
Differently expressed G-lectin genes in *Fragaria vesca* and *F*. *×ananassa* after exposure to either pathogen infection, resistance inducers, or cold treatment. For sources of data see the Methods section. Data are expressed as log_2_ FC (fold-change); NDE, no differential expression. Pc, *Phytophthora cactorum*; Pa, *Podosphaera aphanis*; Bc, *Botrytis cinerea*; BTH, benzothiadiazole; CHI, chitosan; Cold, cold stress; HW, strawberry cv. Hawaii 4 (*F. vesca*); YW, cv. Yellow Wonder 5AF7 (*F. vesca*); BH, cv. Benihoppe (*F. ×ananassa*); AP, cv. Alpine (*F. vesca*); AB, cv. Alba (*F. ×ananassa*); R, root; L, leaf; RF, red fruit; WF, white fruit; h/dpi, hours/days post-inoculation; hpt, hours post-treatment.

Differences in expression of G-lectin genes were found in the interactions of strawberry with *B. cinerea*, *Podosphaera aphanis*, and *Phytophthora cactorum* (Fig. 4). Around 50 G-lectin genes appeared differentially expressed when the roots were infected with *P. cactorum*, with most of them being up-regulated. Several were also found to be up-regulated in response to *Po. aphanis* infection at 8 dpi. Compared to these two pathogens, few G-lectin genes were transcriptionally altered upon *B. cinerea* infection. In addition, some G-lectin genes appeared to be regulated by the plant resistance elicitors benzothiadiazole and chitosan. Interestingly, the homolog of *FaMBL1*, *FveGLP3.7*, was up-regulated upon infection by *B. cinerea*, *Ph. cactorum*, and *Po. aphanis* as well as by benzothiadiazole and chitosan. In terms of abiotic stress, cold stress caused both up- and down-regulation of several G-lectin genes (Fig. 4).

### Functional characterization of *MBL1* in *F.* ×*ananassa*

#### Transgenic lines and their copy numbers

The data reported above together with previously published results (Guidarelli *et al.*, 2014) suggest the importance of the G-type lectin gene family in strawberry defense against pathogens, especially *FveGLP3.7* (homolog of *FaMBL1*). Hence, we used *Agrobacterium*-mediated transformation of octoploid strawberry (*F.×ananassa*, cv. Sveva) (Ma *et al.*, 2021) in order to obtain plants stably overexpressing *FaMBL1*. Three lines (18F6G1, 19F2G1, and 27F8G1) were selected and used for this study. Using ddPCR (Ma *et al.*, 2021), and we determined that the relative copy numbers for 18F6G1, 19F2G1, and 27F8G1 were 6, 2.5, and 1, respectively.

#### *FaMBL1* expression levels in the transgenic lines

**Fig. 5.**
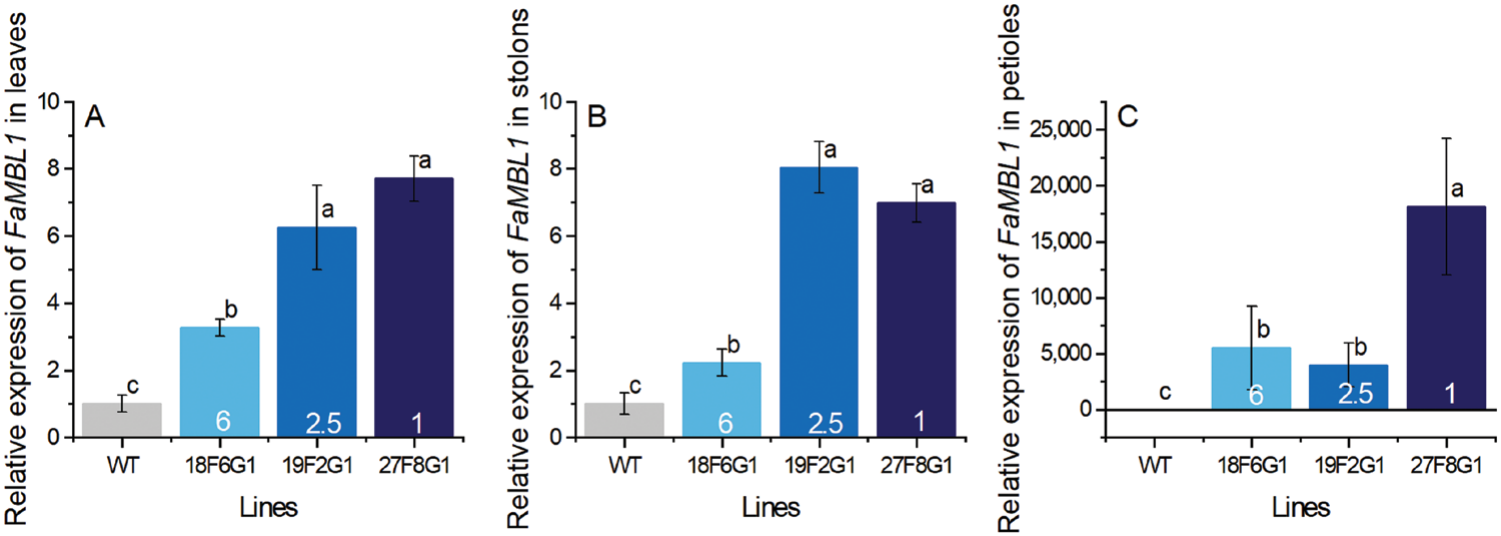
Relative expression of *FaMBL1* in different tissues of wild-type strawberry (*Fragaria* × *ananassa*) and *MBL1*-overexpressing lines. Samples were taken from mature plants of the wild-type (WT) and the three overexpressing lines (18F6G1, 19F2G1, and 27F8G1) and expression was determined by RT qPCR with *EF1a* as the reference gene. Expression is relative to that of the WT, the value of which was set as 1. (A) Leaves, (B) stolons, and (C) petioles. The copy number of *FaMBL1* in each transgenic line is indicated. Data are means (±SD) of three biological replicates. Different letters indicate significant differences among means as determined using one-way ANOVA and Fisher’s LSD test (*P*<0.05).

All three of the selected transgenic lines showed significantly higher *FaMBL1* expression compared to the WT in leaves, stolons, and petioles (Fig. 5). The lowest expression levels were generally found in 18F6G1 and the highest in 27F8G1. *FaMBL1* was barely expressed in the petioles of WT plants (Supplementary Fig. S2) whereas expression was observed in the petioles of the three transgenic lines, thereby indicating the success of the transformation (Fig. 5C).

There was a lack of correlation between the transgene copy number and the *FaMBL1* expression level in the three overexpressing lines. For instance, 27F8G1 (copy number 1) had higher expression levels in all the tissues tested than 18F6G1 (copy number 6; Fig. 5). This suggested that RNAi-mediated suppression of transcript expression might have occurred in those lines containing higher *FaMBL1* copy numbers (Butaye *et al.*, 2005).

#### Phytohormone contents in *FaMBL1*-overexpressing lines

**Fig. 6.**
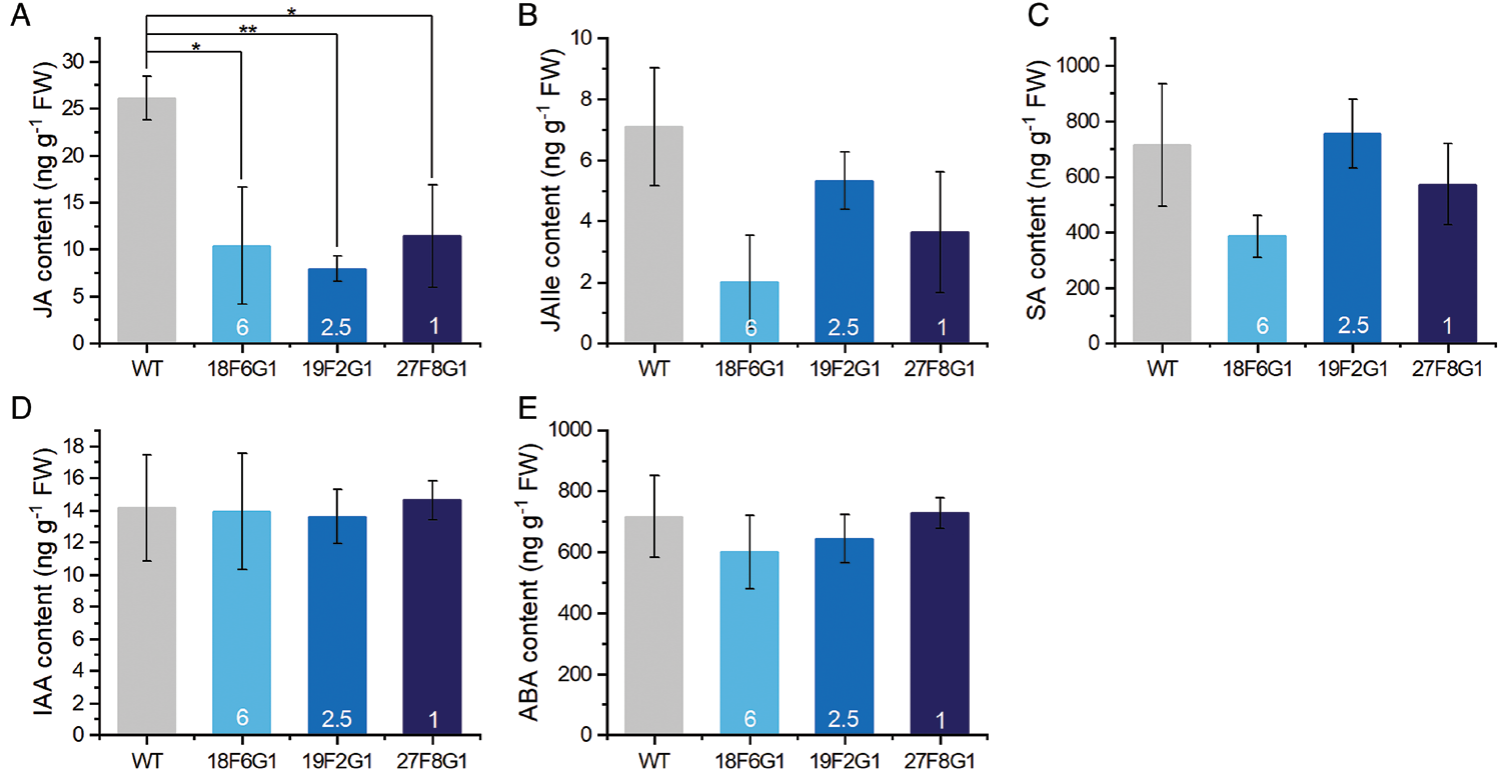
Concentrations of defense-related phytohormones in leaves of wild-type strawberry (*Fragaria* × *ananassa*) and *MBL1*-overexpressing lines. Samples were taken from mature plants of the wild-type (WT) and the three overexpressing lines (18F6G1, 19F2G1, and 27F8G1) and concentrations were determined using UHPLC-MS/MS analysis. (A) Jasmonic acid (JA), (B) jasmonoyl-isoleucine (JA-Ile), (C) salicylic acid (SA), (D) indole-3-acetic acid (IAA), and (E) abscisic acid (ABA). The copy number of *FaMBL1* in each transgenic line is indicated. Data are means (±SD) of three biological replicates. Significant differences between different lines were determined using one-way ANOVA and Fisher’s LSD test: *P<0.05; **P<0.01.

The contents of JA were lower in the overexpressing lines compared to the WT (Fig. 6A), but the effect was reduced and less clear for conjugated JA (Fig. 6B). The contents of IAA, ABA, and SA in the transgenic lines were about the same as those in the WT (Fig. 6C–E) Thus, *FaMBL1* could be directly or indirectly involved in the regulation of JA concentration in strawberry.

#### Response of *FaMBL1*-overexpressing lines to fungal inoculation

**Fig. 7.**
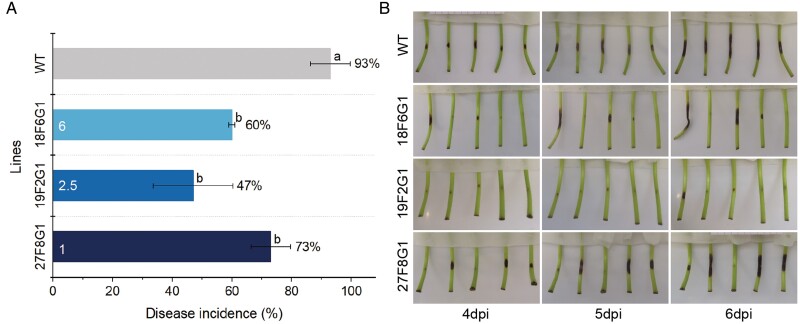
Progression of anthracnose disease on petioles of wild-type strawberry (*Fragaria* × *ananassa*) and *MBL1*-overexpressing lines. Petioles of the wild-type (WT) and the three overexpressing lines (18F6G1, 19F2G1, and 27F8G1) were inoculated with a suspension of conidia of *Colletotrichum fioriniae*. (A) Disease incidence on the petioles at 6 days post-inoculation (dpi). The copy number of *FaMBL1* in each transgenic line is indicated. Petioles with lesions longer than 3 mm were regarded as successfully infected, and incidence was calculated as the number of petioles with symptoms divided by the total number of petioles treated with *C. fioriniae*. Data are means (±SE) of three experiments, each of which consisted five petioles of each line. Different letters indicate significant differences among means as determined using one-way ANOVA and Fisher’s LSD test (*P*<0.05). (B) Images of inoculated petioles of the WT and *FaMBL1*-overexpressing lines at 4–6 dpi in one representative experiment.

We found that *C. fioriniae* became quiescent after infection on strawberry leaves and they appeared symptomless, hence making them unsuitable for evaluation of susceptibility. In contrast, petioles showed uniform and reproducible anthracnose symptoms after inoculation, and hence we used them to examine the responses of the *FaMBL1*-overexpressing lines. The disease incidence was significantly lower in overexpressing lines as compared to the WT at 6 dpi (Fig. 7A), suggesting that the overexpression of *FaMBL1* decreases the susceptibility of strawberry to *C. fioriniae*. Where infection occurred, petioles showed anthracnose symptoms at 4 dpi and this was followed by rapid progress of disease at 5 dpi and 6 dpi, when the symptoms were more apparent (Fig. 7B).

**Fig. 8.**
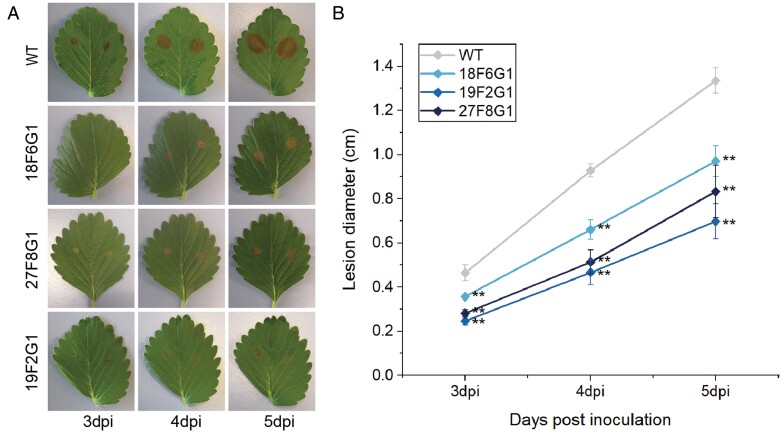
Progression of grey mould disease on detached leaflets of wild-type strawberry (*Fragaria* × *ananassa*) and *MBL1*-overexpressing lines. Leaflets of the wild-type (WT) and the three overexpressing lines (18F6G1, 19F2G1, and 27F8G1) were inoculated with a suspension of conidia of *Botrytis cinera*. (A) Appearance of lesions at 3–5 days post-inoculation (dpi). (B) Increase in lesion size with time. Data are means (±SD) of three experiments, each of which consisted of five leaves separated into leaflets. Significant differences were determined using one-way ANOVA and Fisher’s LSD test (**P<0.01).

Whilst *B. cinerea* can infect both vegetative and reproductive tissues of strawberry, the greatest economic loss due to this pathogen is the result of fruit infection. However, infected vegetative tissues are an important source of inoculum, and improving its resistance is indispensable for the management of *B. cinerea*. Moreover, infection trials using leaves show uniform disease symptoms and reproducible data, which is ideal for evaluation of resistance. Hence, we tested the responses of the *FaMBL1*-overexpressing lines to *B. cinerea* on detached leaflets. We found that necrotic lesions started to be apparent at 3 dpi in both the transgenic lines and the WT (Fig. 8A); however, the disease progression was greater in the WT. The rate increase of lesion size was significantly higher in the WT than in the *FaMBL1*-overexpressing lines (Fig. 8B), indicating that they were less susceptible to *B. cinerea*.

#### Expression of defense-related genes after *B. cinerea* inoculation

**Fig. 9.**
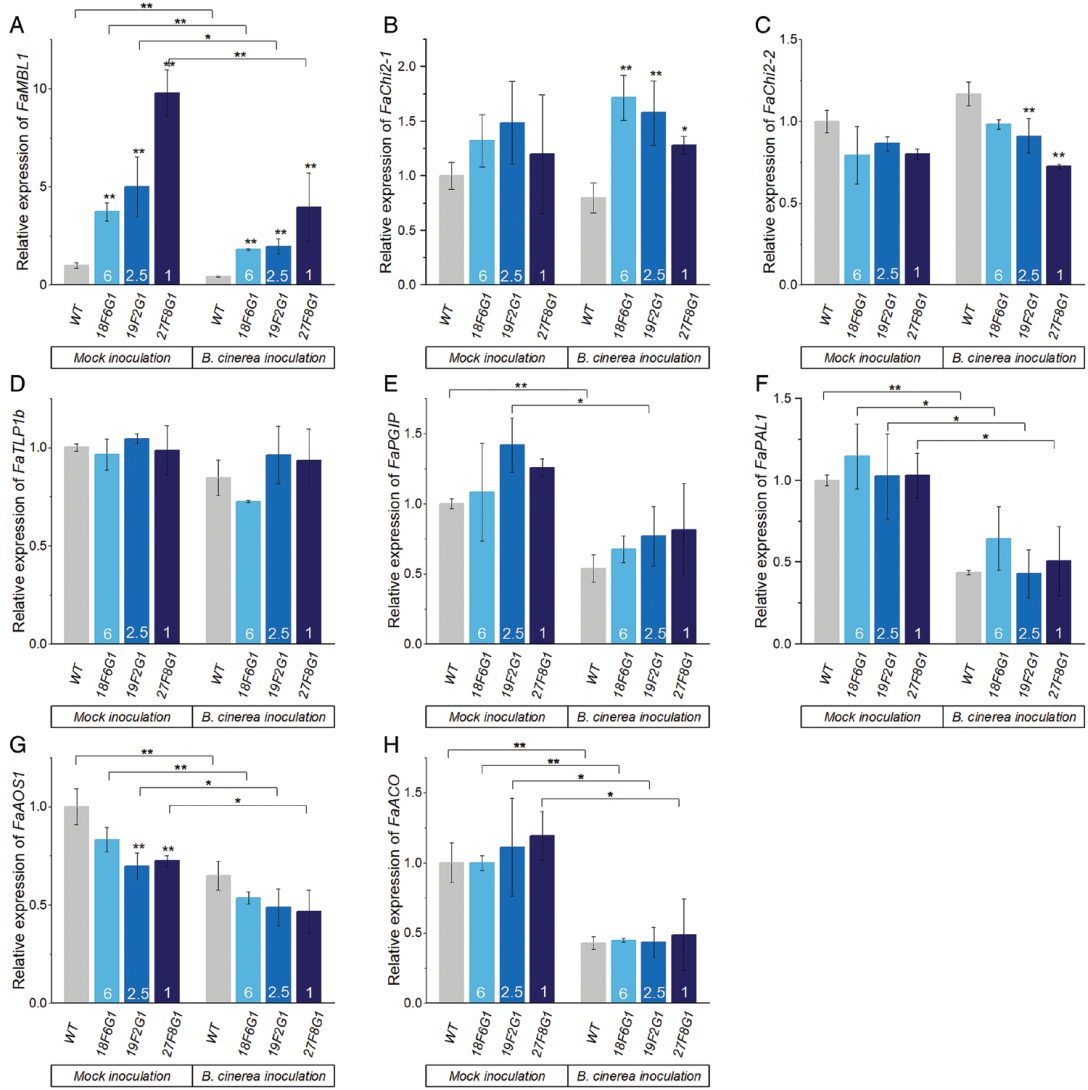
Relative expression of defense-response genes in leaves of wild-type strawberry (*Fragaria* × *ananassa*) and *MBL1*-overexpressing lines challenged with *Botrytis cinerea* at 1 d post-inoculation. Samples were taken from mature plants of the wild-type (WT) and the three overexpressing lines (18F6G1, 19F2G1, and 27F8G1) and expression was determined by RT qPCR with *EF1a* as the reference gene. Expression is relative to that of the WT, the value of which was set as 1. Relative expression of (A) *MANNOSE-BINDING LECTIN 1* (*FaMBL1*), (B) *CLASS II CHITINASE 1* (*FaChi2-1*), (C) *CLASS II CHITINASE 2* (*FaChi2-2*), (D) *THAUMATIN-LIKE PROTEIN 1b* (*FaTLP1b*), (E) *POLYGALACTURONASE-INHIBITING PROTEIN* (*FaPGIP*), (F) *PHENYLALANINE AMMONIA-LYASE 1* (*FaPAL1*), (G) *ALLENE OXIDE SYNTHASE 1* (*FaAOS1*), and (H) *1-AMINOCYCLOPROPANE-1-CARBOXYLATE OXIDASE* (*FaACO*). The copy number of *FaMBL1* in each transgenic line is indicated. Data are means (±SD) of three biological replicates. Significant differences between means were determined using one-way ANOVA and Fisher’s LSD test: *P<0.05; **P<0.01.

To gain insights into the role of *FaMBL1* in strawberry defense against *B. cinerea*, we examined its relative expression together with those of other defense-related genes in the leaves of the overexpressing lines and the WT at 24 h after inoculation. We selected genes that have previously been reported to be induced upon *B. cinerea* infection, namely those encoding CLASS II CHITINASE (*FaChi2-1* and *FaChi2-2*), POLYGALACTURONASE-INHIBITING PROTEIN (*FaPGIP*), PHENYLALANINE AMMONIA-LYASE 1 (*FaPAL1*), THAUMATIN-LIKE PROTEIN 1b (*FaTLP1b*), 1-AMINOCYCLOPROPANE-1-CARBOXYLATE OXIDASE (*FaACO*), AND ALLENE OXIDE SYNTHASE 1 (FaAOS1) (Mehli *et al.*, 2005; Nagpala *et al.*, 2016; Jia *et al.*, 2021; Lee *et al.*, 2021). Whilst *FaMBL1* expression was reduced in both the overexpressing lines and the WT upon *B. cinerea* infection, it remained significantly higher in the overexpressing lines (Fig. 9A). The expression of *FaChi2-1* did not differ among the overexpressing lines and the WT in the absence of inoculation (Fig. 9B); however, after *B. cinerea* infection, its expression was significantly induced only in the overexpressing lines (Fig. 9B). The overexpressing lines 19F2G1 and 27F8G1 showed significant down-regulation of *FaChi2-2* compared with the WT in infected plants (Fig. 9C) and significant down-regulation of *FaAOS1* compared with the WT in the absence of infection (Fig. 9G). No other genes showed any significant differences in expression between the *FaMBL1*-overexpressing lines and the WT in the absence of infection. In contrast, in the presence of *B. cinerea* the expression of the defense-related genes *FaPGIP*, *FaPAL1*, *FaAOS1* (involved in JA synthesis), and *FaACO* (involved in ET synthesis) was down-regulated in both the overexpressing and WT plants (Fig. 9E–H).

## Discussion

Anthracnose (*Colletotrichum acutatum* species complex) and grey mould (*Botrytis cinerea*) are two of the most destructive strawberry fungal diseases. Increasing plant resistance is one of the most sustainable and effective strategies for management; however, the high levels of heterozygosity and polyploidy in strawberry increase the complexity of applying traditional breeding methods (Nehra *et al.*, 1990; Limera *et al.*, 2017; Mezzetti *et al.*, 2018), and hence the importance of genetic transformation techniques for investigating resistance genes.

G-type lectins are reported to play roles in plant defense against biotic and abiotic stresses (Ghequire *et al.*, 2012; Siripipatthana *et al.*, 2015). In woodland strawberry, *F. vesca*, we found 133 genes belonging to the G-lectin family (Fig. 1). The FveG-lectin proteins encoded by these genes showed multiple domain arrangements, which creates a fairly high degree of protein diversity and provides flexible adaptation to changing environments (Kersting *et al.*, 2012). According to the transcriptome data, many G-lectin genes are actively expressed in different tissues at different developmental stages of strawberries (Fig. 3; Supplementary Fig. S1). Moreover, many G-lectins in strawberry actively respond to pathogens and elicitors, and some appear to respond to biotic stresses. Notably, red fruits infected with *B. cinerea* have been found to show different expression profiles of G-lectin genes at 24 h post-infection (hpi; Fig. 4). In particular, *FveGLP3.7*, the *FaMBL1* homolog, is up-regulated in response to *B. cinerea* in the octoploid red fruit of *F.* ×*ananassa* cv. Benihoppe (BH in Fig. 4) but not in diploid fruit of cv. Alpine (AP). On the other hand, we found that expression of *FaMBL1* was down-regulated in leaves of octoploid cv. Sveva infected with *B. cinerea* at 24 hpi (Fig. 9A), suggesting that the regulation of this gene in response to infection is not only cultivar-specific but also dependent on the tissue type. The resistance to *B. cinerea* that we observed in the leaves of transgenic plants overexpressing *FaMBL1* (Fig. 8) could be due to signaling pathways downstream of *FaMBL1* that are still to be elucidated. Given that the homolog of *FaMBL1*, *FveGLP3.7*, is up-regulated upon infection by the biotroph *Podopshaera aphanis* and the hemibiotroph *Phytophtora cactorum* (Fig.4), which are fungal pathogens with different infection strategies to *B. cinerea*, it is most likely that *FaMBL1* acts as a key regulator in strawberry response to pathogens, with different expression pathways depending on the specific biotic stress (Guidarelli *et al.*, 2014).

Given the potential of FaMBL1 in defense, we have previously produced genetically transformed *F.* ×*ananassa* plants overexpressing *FaMBL1* to confirm its role as defense protein (Ma *et al.*, 2021). We obtained three overexpressing lines with different copy numbers and increased *FaMBL1* expression in leaves, stolons, and petioles compared to the WT (Fig. 5). The differences in insertion copy number were not reflected in the expression levels in the different overexpressing lines. Thus, line 18F6G1 that possesses six copies had lower expression of *FaMBL1* than line 27F8G1 that has only one copy. This is possibly due to the epigenetic silencing mechanism activated by plants in response to multiple transgene copies (Vaucheret *et al.*, 1998). Notably, *FaMBL1* was not expressed in petioles of the WT (Fig. 5C; Supplementary Fig. S2), whereas ectopic expression was observed in the petioles of transgenic plants driven the by *35S* promoter. The decreased susceptibility to *C. fioriniae* observed in the petioles of transgenic plants therefore further support the role of *FaMBL1* in resistance to anthracnose disease.

Plant hormones play important roles in modulating plant resistance and susceptibility to pathogens (Robert-Seilaniantz *et al.*, 2011). G-type lectins in other species have been reported to be involved in phytohormone-related defense pathways. For example, the pepper G-lectin gene *CaMBL1* has been found to be indispensable for the defense response against *Xanthomonas campestris* pv *vesicatoria*, with transient expression inducing the accumulation of SA and the activation of defense-related genes (Hwang and Hwang, 2011). In this study, the JA content was reduced in the *FaMBL1*-overexpressing lines compared to the WT (Fig. 6A), suggesting that *FaMBL1* might participate in the modulation of JA concentration. JA is a hormone that is well known for its role as an elicitor of plant responses to pathogens, wounding, and herbivores (Antico *et al.*, 2012). A number of studies have consistently reported that JA-dependent signaling can trigger the expression of defense-related effector genes and that JA-deficient mutants display increased susceptibility to infection by necrotrophic fungi (see review by Antico *et al.*, 2012). However, there are also several studies that have shown that some mutations causing deficiency in JA biosynthesis display increased resistance to some necrotrophic fungi (Antico *et al.*, 2012). Taken together, these observations reveal the complexity of the JA-dependent regulation of plant responses.

The decreased JA production that we observed in the *FaMBL1*-overexpressing lines might ultimately be related to the resistance to fungal pathogens that we observed; however, the data that we obtained are not sufficient to confirm this hypothesis. Further experiments are needed to establish a relationship between *FaMBL1* expression and JA-mediated defense responses, using exogenous applications of inducers or inhibitors and quantification of the hormones in infected tissues.

Infection with *B. cinerea* has been reported to induce the expression of *CLASS II CHITINASE* (*FaChi2-1* and *FaChi2-2*), *POLYGALACTURONASE-INHIBITING PROTEIN* (*FaPGIP*), *PHENYLALANINE AMMONIA-LYASE 1* (*FaPAL1*), *THAUMATIN-LIKE PROTEIN 1b* (*FaTLP1b*), *1-AMINOCYCLOPROPANE-1-CARBOXYLATE OXIDASE* (*FaACO*), and *ALLENE OXIDE SYNTHASE 1* (*FaAOS1*) and to contribute to strawberry resistance against this pathogen (Mehli *et al.*, 2005; Nagpala *et al.*, 2016; Jia *et al.*, 2021; Lee *et al.*, 2021). After strawberry infection with *C. acutatum*, uncoupling between SA and JA accumulation and the induction of important SA- and JA-related genes has also been reported, including *PATHOGENESIS-RELATED PROTEIN 1* (*FaPR1-1*), *LIPOXYGENASE-2* (FaLOX2), *JASMONATE RESISTANT-1* (FaJAR1), and *PLANT DEFENSIN-1* (FaPDF1) (Amil-Ruiz *et al.*, 2016). We assessed the expression of some of these genes in the leaves of the *FaMBL*-overexpressing lines at 24 h after *B. cinerea* inoculation and found that they showed higher expression of *FaChi2-1* than the WT (Fig. 9B). *FaChi* is one of the most abundant classes of strawberry pathogenesis-related genes and has hydrolytic activity against fungal cell walls (Amil-Ruiz *et al.*, 2011). *FaChi2-1* has been found to be involved in defense responses against both anthracnose (Tortora *et al.*, 2012) and grey mould (Mehli *et al.*, 2005). Overall, the higher expression of *FaMBL1* in the overexpressing lines can be associated with the higher expression of *FaChi2-1* at the early stage of infection by *B. cinerea*. The rice G-type lectin gene, *OslecRK*, which is consistently associated with resistance to *M. grisea*, *Xanthomonas oryzae*, and brown planthoppers, influences the expression of defense-related genes such as *PR1a*, lipoxygenase, and chalcone synthase (Cheng *et al.*, 2013). Interestingly, we found that the expression of *FaAOS1*, a key enzyme in JA biosynthesis, was significantly lower in the 19F2G1 and 27F8G1 transgenic lines compared to the WT in the absence of infection (Fig. 9G), and this could be related to the lower JA contents in the overexpressing lines compared to the WT (Fig. 6A). In contrast, in 18F6G1, where the level of expression of *FaMBL1* was lower with respect to the other lines (Fig. 5), the expression of *FaAOS1* was not significantly different to that the WT (Fig. 9G), supporting the hypothesis of an involvement of *FaMBL1* in downstream regulation. Similarly, the expression of *FaChi2-2* was not significantly different in 18F6G1 (Fig. 9C). More studies are needed to clarify the involvement of FaMBL1 in the regulation of downstream expression of defense genes.

In conclusion, G-type lectins form a large gene family in *F. vesca* and have exploitable potential for strawberry defense against biotic stresses. We found that transgenic octoploid strawberries overexpressing *FaMBL1* were less susceptible to the fungal diseases anthracnose and grey mould. The association that we observed between *FaMBL1* and JA-dependent signaling pathways should be further investigated in order to obtain more evidence of the defense role of *FaMBL1*. This gene could be used in future trials for improving resistance in economically important strawberry cultivars through full transgenic, cisgenic, or genome-editing approaches.

## Supplementary data

The following supplementary data are available at *JXB* online.

Fig. S1. Expression profiles of G-type lectin genes in different tissues of *Fragaria vesca* and at different developmental stages.

Fig. S2. Relative expression of *FaMBL1* in different tissues of wild-type *Fragaria* × *ananassa*.

Table S1. List of primers used in this study.

Table S2. Proposed nomenclature for G-type lectin genes in *Fragaria vesca*.

erac396_suppl_Supplementary_Material

## Data Availability

All data supporting the findings of this study are available within the paper and within its supplementary materials published online.

## Abbreviations

ABA: abscisic acid
GNA: *Galanthus nivalis* agglutinin-related lectin
G-LecK: G-type lectin kinase
G-LecP: G-type lectin protein
G-LecRK: G-type lectin receptor kinase
G-LecRP: G-type lectin receptor protein
IAA: indole-3-acetic acid
JA: jasmonic acid
JA-Ile: jasmonoyl-isoleucine
PAN: Pan-apple
PDA: potato dextrose agar
PK: protein kinase
SA: salicylic acid
TM: transmembrane.
