# Supplementary material for: Functional characterization of MANNOSE-BINDING LECTIN 1, a G-type lectin gene family member, in response to fungal pathogens of strawberry
Source: J Exp Bot. 2022 Oct 11;74(1):149–61. doi: 10.1093/jxb/erac396 (PMC9786840; doi:10.1093/jxb/erac396)
Supplement: erac396_suppl_Supplementary_Material [file erac396_suppl_supplementary_material.pdf]

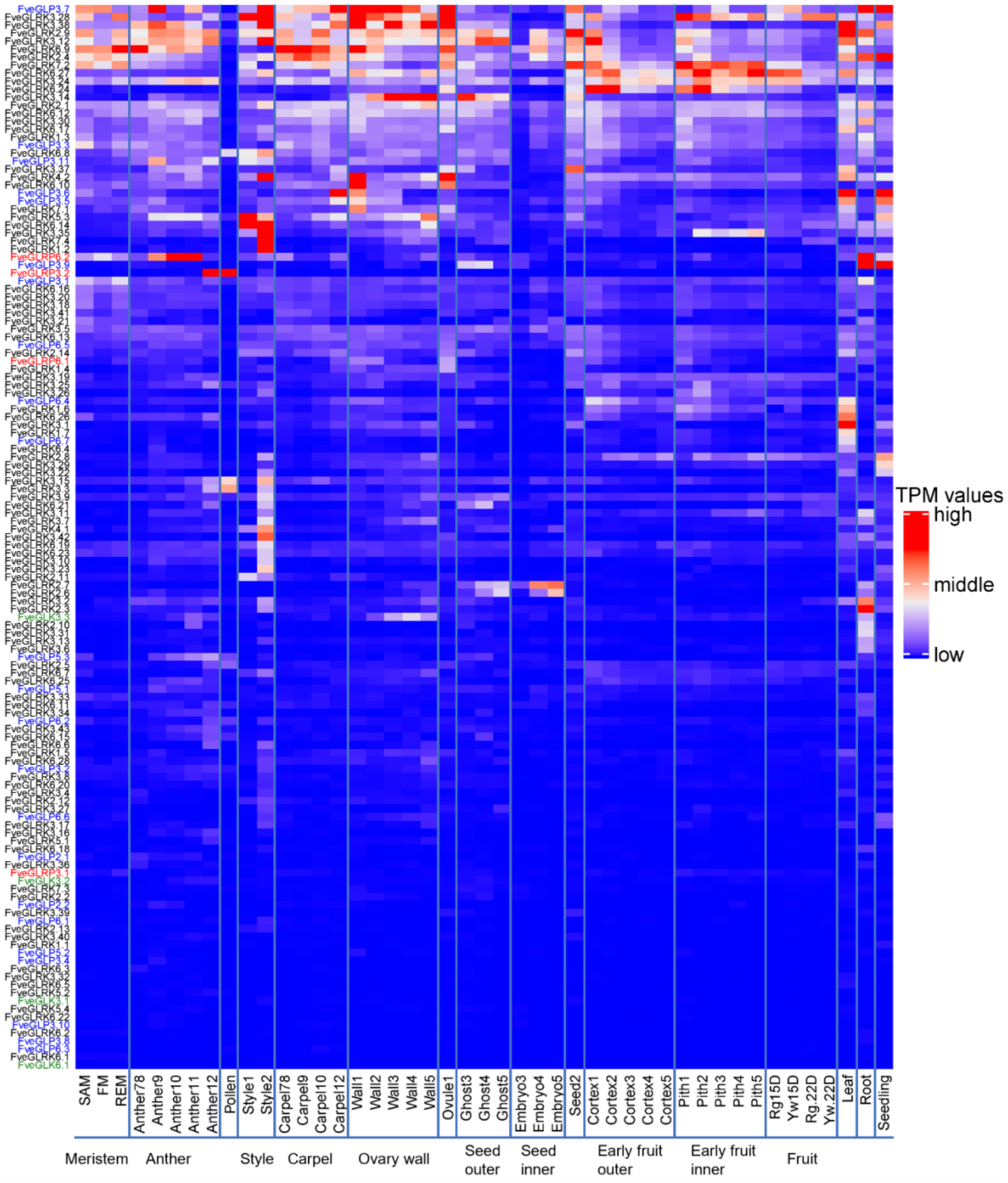

Fig. S1. Expression profiles of G-type lectin genes in *Fragaria vesca* tissues and developmental stages. The different types of G-lectin genes are reported on the left in different colors: G-type lectin receptor kinases (G-LecRKs) in black, G-type lectin proteins (G-LecPs) in blue, G-type

lectin receptor proteins (G-LecRPs) in red, and G-type lectin kinases (G-LecKs) in green. The expression levels are indicated by TPM (transcript per million reads) and the TPM value for each gene is scaled before constructing the heatmap. SAM: shoot apical meristem; FM: flower meristem; REM: receptacle meristem; Anther78/9/10/11/12: stage 7-8/9/10/11/12 anther; Style1/2: stage1/2 style; Carpel78/9/10/12: stage 7-8/9/10/12 carpel; Wall1/2/3/4/5: stage 1/2/3/4/5 ovary wall; Ovule1: stage 1 ovule; Ghost3/4/5: stage 3/4/5 endosperm and seed coat; Embryo3/4/5: stage 3/4/5 embryo; Cortex1/2/3/4/5: outer part of the early fruit at stage 1/2/3/4/5; Pith1/2/3/4/5: inner part of the early fruit at stage 1/2/3/4/5; Rg.15D: green fruit stage of Ruegen; Yw.15D: green fruit stage of Yellow Wonder 5AF7; Rg.22D: white fruit stage of Ruegen; Yw.22D: white fruit stage of Yellow Wonder 5AF7.

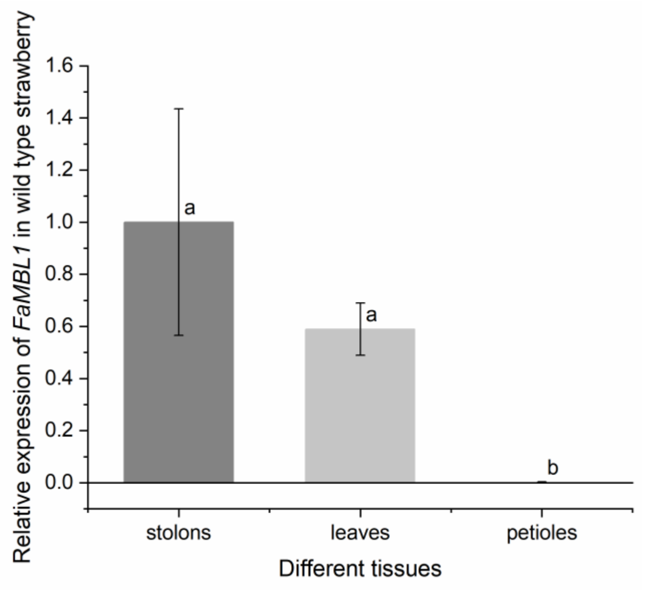

Fig. S2. Relative expression of *FaMBL1* in different tissues of wild type strawberry (*Fragaria* × *ananassa*). All values were normalized to the expression level of the *elongation factor 1α* housekeeping gene. The expression of *FaMBL1* gene in stolons was regarded as 1. Each value is an average of three biological replicates with its standard deviation. Means with the same letter are not significantly different at $P < 0.05$.

Table S1. List of primer name and primer sequence with their description

Primer description	Primer names	Primer sequences from 5' to 3'
RT-PCR *FaMBL1* gene	FaMBL1_CUR fw	AAACCAACACGGCCAATAAG
	FaMBL1_CUR rev	GTCTGTCGGGTAGTCGAAGC
RT-PCR reference gene	FaEF1a fw	TGGATTTGAGGGTGACAACATGA
	FaEF1a rev	GTATACATCCTGAAGTGGTAGTCGGAGG
FaPAL1	FaPAL1 fw	TGGACTACGGCTTCAAAGGT
	FaPAL1 rev	GACATCTTGGTTGTGTTGCTC
FaPGIP	FaPGIP fw	ATCTCACAGGTCCCATCCAG
	FaPGIP rev	GCTGAGGAAGTCAGGGACTG
FaAOS1	FaAOS1 fw	AGGTTAAAGAAGGCGAGGTGTT
	FaAOS1 rev	GAGGACCGTTAGACCAAAGCA
FaChi2-1	FaChi2-1 fw	AAGCCCTCTTGTCACGATGT
	FaChi2-1 rev	TCGAGTCCACCGTTGATGAT
FaChi2-2	FaChi2-2 fw	ATGTG GGCGT GGACA AGATA
	FaChi2-2 rev	AACAG TCCAA GTTGT CCCCA
FaACO	FaACO fw	AGGTTAAAGAAGGCGAGGTGTT
	FaACO rev	GAGGACCGTTAGACCAAAGCA
FaTLP1b	FaTLP1b fw	GTGCATCACTTCTTCCAAGT
	FaTLP1b rev	CAAACCTGCTAGCAGTGAAG

Table S2. Proposed nomenclature for G-type lectin genes in *Fragaria vesca*

Classification	Gene ID	New Name	Classification	Gene ID	New Name
G-type lectin receptor kinase	FvH4_1g03780	FveGLRK1.1	G-type lectin receptor kinase	FvH4_5g31680	FveGLRK5.2
	FvH4_1g04840	FveGLRK1.2		FvH4_5g31930	FveGLRK5.3
	FvH4_1g16211	FveGLRK1.3		FvH4_5g32570	FveGLRK5.4
	FvH4_1g23370	FveGLRK1.4		FvH4_6g00257	FveGLRK6.1
	FvH4_1g23380	FveGLRK1.5		FvH4_6g00270	FveGLRK6.2
	FvH4_1g23390	FveGLRK1.6		FvH4_6g07960	FveGLRK6.3
	FvH4_1g23400	FveGLRK1.7		FvH4_6g12870	FveGLRK6.4
	FvH4_2g12390	FveGLRK2.1		FvH4_6g12880	FveGLRK6.5
	FvH4_2g14250	FveGLRK2.2		FvH4_6g12890	FveGLRK6.6
	FvH4_2g26490	FveGLRK2.3		FvH4_6g12930	FveGLRK6.7
	FvH4_2g29050	FveGLRK2.4		FvH4_6g20800	FveGLRK6.8
	FvH4_2g29070	FveGLRK2.5		FvH4_6g26380	FveGLRK6.9
	FvH4_2g29542	FveGLRK2.6		FvH4_6g26420	FveGLRK6.10
	FvH4_2g29543	FveGLRK2.7		FvH4_6g26450	FveGLRK6.11
	FvH4_2g29544	FveGLRK2.8		FvH4_6g29821	FveGLRK6.12
	FvH4_2g29545	FveGLRK2.9		FvH4_6g29840	FveGLRK6.13
	FvH4_2g29560	FveGLRK2.10		FvH4_6g31370	FveGLRK6.14
	FvH4_2g33830	FveGLRK2.11		FvH4_6g44063	FveGLRK6.15
	FvH4_2g33840	FveGLRK2.12		FvH4_6g44064	FveGLRK6.16
	FvH4_2g33850	FveGLRK2.13		FvH4_6g44100	FveGLRK6.17
	FvH4_2g33870	FveGLRK2.14		FvH4_6g44106	FveGLRK6.18
	FvH4_3g03230	FveGLRK3.1		FvH4_6g44107	FveGLRK6.19
	FvH4_3g03231	FveGLRK3.2		FvH4_6g44108	FveGLRK6.20
	FvH4_3g03240	FveGLRK3.3		FvH4_6g44109	FveGLRK6.21
	FvH4_3g03242	FveGLRK3.4		FvH4_6g44140	FveGLRK6.22
	FvH4_3g03243	FveGLRK3.5		FvH4_6g44190	FveGLRK6.23
	FvH4_3g03301	FveGLRK3.6		FvH4_6g44243	FveGLRK6.24
	FvH4_3g03310	FveGLRK3.7		FvH4_6g44244	FveGLRK6.25
	FvH4_3g03320	FveGLRK3.8		FvH4_6g44245	FveGLRK6.26
	FvH4_3g03350	FveGLRK3.9		FvH4_6g44310	FveGLRK6.27
	FvH4_3g03370	FveGLRK3.10		FvH4_6g51830	FveGLRK6.28
	FvH4_3g03390	FveGLRK3.11		FvH4_7g00200	FveGLRK7.1
	FvH4_3g03410	FveGLRK3.12		FvH4_7g14760	FveGLRK7.2
	FvH4_3g03420	FveGLRK3.13		FvH4_7g19680	FveGLRK7.3
	FvH4_3g03430	FveGLRK3.14		FvH4_7g30670	FveGLRK7.4
	FvH4_3g03431	FveGLRK3.15	G-type lectin protein	FvH4_2g05942	FveGLP2.1
	FvH4_3g03432	FveGLRK3.16		FvH4_2g24770	FveGLP2.2

	FvH4_3g03433	FveGLRK3.17		FvH4_3g03322	FveGLP3.1
	FvH4_3g03450	FveGLRK3.18		FvH4_3g03340	FveGLP3.2
	FvH4_3g03451	FveGLRK3.19		FvH4_3g03435	FveGLP3.3
	FvH4_3g03461	FveGLRK3.20		FvH4_3g15150	FveGLP3.4
	FvH4_3g03481	FveGLRK3.21		FvH4_3g18370	FveGLP3.5
	FvH4_3g03482	FveGLRK3.22		FvH4_3g18371	FveGLP3.6
	FvH4_3g03501	FveGLRK3.23		FvH4_3g18380	FveGLP3.7
	FvH4_3g03502	FveGLRK3.24		FvH4_3g18382	FveGLP3.8
	FvH4_3g03520	FveGLRK3.25		FvH4_3g18383	FveGLP3.9
	FvH4_3g03521	FveGLRK3.26		FvH4_3g18410	FveGLP3.10
	FvH4_3g03560	FveGLRK3.27		FvH4_3g21271	FveGLP3.11
	FvH4_3g03590	FveGLRK3.28		FvH4_5g04270	FveGLP5.1
	FvH4_3g06140	FveGLRK3.29		FvH4_5g04310	FveGLP5.2
	FvH4_3g15080	FveGLRK3.30		FvH4_5g31690	FveGLP5.3
	FvH4_3g15120	FveGLRK3.31		FvH4_6g00300	FveGLP6.1
	FvH4_3g15130	FveGLRK3.32		FvH4_6g12332	FveGLP6.2
	FvH4_3g15690	FveGLRK3.33		FvH4_6g12920	FveGLP6.3
	FvH4_3g15930	FveGLRK3.34		FvH4_6g44062	FveGLP6.4
	FvH4_3g21270	FveGLRK3.35		FvH4_6g44101	FveGLP6.5
	FvH4_3g21310	FveGLRK3.36		FvH4_6g44242	FveGLP6.6
	FvH4_3g21320	FveGLRK3.37		FvH4_6g44260	FveGLP6.7
	FvH4_3g21400	FveGLRK3.38	G-type lectin kinase	FvH4_3g03241	FveGLK3.1
	FvH4_3g43401	FveGLRK3.39		FvH4_3g03300	FveGLK3.2
	FvH4_3g43402	FveGLRK3.40		FvH4_3g15980	FveGLK3.3
	FvH4_3g43403	FveGLRK3.41		FvH4_6g44240	FveGLK6.1
	FvH4_3g43440	FveGLRK3.42	G-type lectin receptor protein	FvH4_3g03581	FveGLRP3.1
	FvH4_3g43710	FveGLRK3.43		FvH4_3g15090	FveGLRP3.2
	FvH4_4g02170	FveGLRK4.1		FvH4_6g10470	FveGLRP6.1
	FvH4_4g33230	FveGLRK4.2		FvH4_6g17930	FveGLRP6.2
	FvH4_5g04350	FveGLRK5.1			

Table S3. MIQE checklist for authors, reviewers, and editors.

ITEM TO CHECK	IMPORTANCE	CHECKLIST
EXPERIMENTAL DESIGN		
Definition of experimental and control groups	E	Yes
Number within each group	E	N/A
Assay carried out by core lab or investigator's lab?	D	Yes
Acknowledgement of authors' contributions	D	Yes
SAMPLE		
Description	E	Yes
Volume/mass of sample processed	D	Yes
Microdissection or macrodissection	E	N/A
Processing procedure	E	Yes
If frozen - how and how quickly?	E	Yes
If fixed - with what, how quickly?	E	N/A
Sample storage conditions and duration (especially for FFPE samples)	E	Yes
NUCLEIC ACID EXTRACTION		
Procedure and/or instrumentation	E	Yes
Name of kit and details of any modifications	E	Yes
Source of additional reagents used	D	N/A
Details of DNase or RNAse treatment	E	Yes
Contamination assessment (DNA or RNA)	E	N/A
Nucleic acid quantification	E	Yes
Instrument and method	E	Yes
Purity (A260/A280)	D	N/A
Yield	D	N/A
RNA integrity method/instrument	E	Yes
RIN/RQI or Cq of 3' and 5' transcripts	E	N/A
Electrophoresis traces	D	Yes
Inhibition testing (Cq dilutions, spike or other)	E	N/A
REVERSE TRANSCRIPTION		
Complete reaction conditions	E	Yes
Amount of RNA and reaction volume	E	Yes
Priming oligonucleotide (if using GSP) and concentration	E	N/A
Reverse transcriptase and concentration	E	Yes
Temperature and time	E	Yes
Manufacturer of reagents and catalogue numbers	D	N/A
Cqs with and without RT	D*	N/A
Storage conditions of cDNA	D	Yes
qPCR TARGET INFORMATION		
If multiplex, efficiency and LOD of each assay.	E	N/A
Sequence accession number	E	Yes
Location of amplicon	D	N/A
Amplicon length	E	Yes
In silico specificity screen (BLAST, etc)	E	N/A
Pseudogenes, retropseudogenes or other homologs?	D	N/A
Sequence alignment	D	N/A
Secondary structure analysis of amplicon	D	Yes
Location of each primer by exon or intron (if applicable)	E	N/A
What splice variants are targeted?	E	N/A
qPCR OLIGONUCLEOTIDES		
Primer sequences	E	Yes

RTPrimerDB Identification Number	D	N/A
Probe sequences	D**	N/A
Location and identity of any modifications	E	N/A
Manufacturer of oligonucleotides	D	N/A
Purification method	D	N/A
qPCR PROTOCOL		
Complete reaction conditions	E	Yes
Reaction volume and amount of cDNA/DNA	E	Yes
Primer, (probe), Mg++ and dNTP concentrations	E	Yes
Polymerase identity and concentration	E	Yes
Buffer/kit identity and manufacturer	E	Yes
Exact chemical constitution of the buffer	D	N/A
Additives (SYBR Green I, DMSO, etc.)	E	Yes
Manufacturer of plates/tubes and catalog number	D	N/A
Complete thermocycling parameters	E	Yes
Reaction setup (manual/robotic)	D	N/A
Manufacturer of qPCR instrument	E	Yes
qPCR VALIDATION		
Evidence of optimisation (from gradients)	D	N/A
Specificity (gel, sequence, melt, or digest)	E	Yes
For SYBR Green I, Cq of the NTC	E	Yes
Standard curves with slope and y-intercept	E	Yes
PCR efficiency calculated from slope	E	Yes
Confidence interval for PCR efficiency or standard error	D	N/A
r2 of standard curve	E	Yes
Linear dynamic range	E	Yes
Cq variation at lower limit	E	N/A
Confidence intervals throughout range	D	N/A
Evidence for limit of detection	E	N/A
If multiplex, efficiency and LOD of each assay.	E	Yes
DATA ANALYSIS		
qPCR analysis program (source, version)	E	Yes
Cq method determination	E	Yes
Outlier identification and disposition	E	Yes
Results of NTCs	E	Yes
Justification of number and choice of reference genes	E	Yes
Description of normalisation method	E	Yes
Number and concordance of biological replicates	D	Yes
Number and stage (RT or qPCR) of technical replicates	E	Yes
Repeatability (intra-assay variation)	E	N/A
Reproducibility (inter-assay variation, %CV)	D	N/A
Power analysis	D	N/A
Statistical methods for result significance	E	Yes
Software (source, version)	E	Yes
Cq or raw data submission using RDML	D	N/A
